# Annotations

**Published:** 1892-10-15

**Authors:** 


### Annotations.

**What to do with Female Drunkards.**

Now that the period of excitement is over we may soberly consider what the *Daily Telegraph* calls our "National Shame." The fact of an alarming prevalence of drunkenness among women cannot be denied. Poor women especially seem to be giving themselves up to this dreadful degradation and ruin in increasing numbers. Is there any widespread cause for this? If there be, those of us who feel the responsibility that rests upon our nation should try to find it out. There are causes, most potent causes, which are known to medical men. The chief of these causes are summed up in a few words by the *British Medical Journal*, and much would be gained if intelligent men and women would read, and really lay to heart what the *Journal* says upon the point. Here is the pith and marrow of it: "The frequent systemic perturbation which involves acute suffering to so many women from functional crises peculiar to the sex is responsible for probably the largest number of cases of inebriety arising from any single cause. Transmission of the inebriate diathesis, of some other exchangeable neurosis, and of mental instability comes next, and, indeed, adds to the inebriety generating influence of the former source. Mental and physical overwork, household and family worry, the neurasthenia of other diseases, of too frequent parturition and of accident, account for a goodly proportion. Idleness, want of steady occupation or pursuit, which would distract the individual from that *ennui* and depression which invite the deceptive solace of anæsthetics, with other predisposing and exciting causes, are the starting point to an inebriate career of not a few. Selfish indulgence to every passing whim, the non-cultivation of inhibition and thought also swell the roll of causes which render women an easy prey to the delusive and transitory lethe of intoxication, and break down the will." This is a painfully formidable array of causes, and almost makes the physiologist and moralist despair. There are manifestly two lines of activity upon which those who strive to bring about a better state of things must move. In the first place, the physical and mental burdens of women, especially of the working classes, must be lightened; those burdens are now well-nigh unendurable. In the second place, the moral and religious elements of the female character must be strengthened. The question is one of national importance. It will need the best efforts of the best intellects we have to deal with it. The responsibility rests mainly upon three classes — statesmen, religious teachers, and medical men. The crisis is urgent. A special organisation of the most competent members of all these three classes is immediately demanded. Who will bring the question to this definite head?

**Vivisection at the Church Congress.**

Mr. Victor Horsley spoke his mind with much freedom to the members of the Church Congress on the subject of vivisection. He used—in scriptural phrase—"great plainness of speech." He acted wisely and worthily in so doing. Mr. Horsley proved two things very convincingly: First, that medical men, the only really competent judges on the question, are practically unanimous in their decision that vivisection has been, and is, of great service in the arts of medicine and surgery; and secondly, that the opponents of it have been uncandid in their arguments, and abusive in their language. The real question at issue—the philosophico-moral question—whether, notwithstanding its admitted value, vivisection is morally justifiable, was not touched. It is evident now that if it had been argued it could not have been argued properly. Personal feeling ran too high on both sides. We cannot but express the conviction that for this personal bitterness and rancour the anti-vivisectionists, notably Miss Frances Power Cobbe, Bishop Barry, and Canon Wilberforce, have been chiefly responsible. They have not conducted the controversy in either a Christian or a scientific spirit. In refusing to examine vivisections and the work of vivisectors personally, and in taking their facts largely at secondhand, they have been demonstratively unscientific: and in calling vivisectors "inhuman devils," and stigmatising their work as "abominable sin," they have still more patently transgressed the great law of Christian charity. These are grave mistakes, and, indeed, grave offences. Candour and charity, in common English, fairplay and kind feeling, are of the very elements of Christianity, as they are of the English character. Nothing whatever can justify the throwing of them away. Miss Cobbe, Bishop Barry, and Canon Wilberforce are quite entitled to object to vivisection, and to try to rouse public opinion against it. But when they resort to unfairness and abuse, they injure their own cause, and still more their characters. The matter at present stands thus: The voice of the medical profession is almost unanimous in favour of vivisection under humane restrictions; on the other hand, a great many excellent people are opposed to it. Then, let both sides reason out the question fairly from every standpoint, and let the public decide the issue—as it will in due time. There is really nothing else to be done. We venture to ask both sides to put down their brickbats, and to take up the weapons of sound logic and a kindly humanity.

**Lord Tennyson's Kindness to Nurses.**

It seems as if the reading public could hardly hear too much of him who was the last to be buried in Poets' Corner. He had won the confidence and the affection of his fellow-countrymen, even as he had compelled their admiration. Our little contribution to the cairn which is being raised to his memory is trifling, but it is not without significance. When the daughter of Princess Christian was married last year, the nurses of the Royal National Pension Fund desired to give her a wedding present, and after much deliberation it was decided that the works of Tennyson, bound in white vellum, would be an appropriate, as it proved to be a welcome gift. The volumes were printed on hand-made paper and beautifully bound, the publisher giving them as a free gift, and the binder charging only for work and cost of material. Lord Tennyson, who had required the services of a nurse for a long time in 1889-90, was asked if he would inscribe a few original lines in the first volume, with his autograph. Although, as is well known, he was not easily induced to give rein to his muse at the bidding of another, he very kindly consented to do as he was asked; and the following lines, with his autograph, were inscribed in the first volume of the series now in the possession of the young princess. They are quoted from memory:—

> Take, lady, what your loyal nurses give,
> Their full, God bless you, with this book of song;
> And may the life which heart in heart you live,
> With him you love, be cloudless, and be long.

It is perhaps a trifle, but it is an added testimony to the gentleness which was a part of the dead poet's character. Nurses, and no doubt other readers, will be glad to be reminded of this pleasing circumstance. It may not be out of place to add that the two hundred pounds which Lord Tennyson paid to his nurse, Miss Emma Durham, for long and faithful services, found its way, intact, by that nurse's generosity, into the treasury of the Junius S. Morgan Benevolent Fund for Poor Nurses.

